# Referential shift in Nicaraguan Sign Language: a transition from lexical to spatial devices

**DOI:** 10.3389/fpsyg.2014.01540

**Published:** 2015-01-09

**Authors:** Annemarie Kocab, Jennie Pyers, Ann Senghas

**Affiliations:** ^1^Department of Psychology, Harvard UniversityCambridge, MA, USA; ^2^Department of Psychology, Wellesley CollegeWellesley, MA, USA; ^3^Department of Psychology, Barnard College, Columbia UniversityNew York, NY, USA

**Keywords:** referential shift, narratives, spatial language, sign language, language creation

## Abstract

Even the simplest narratives combine multiple strands of information, integrating different characters and their actions by expressing multiple perspectives of events. We examined the emergence of *referential shift* devices, which indicate changes among these perspectives, in Nicaraguan Sign Language (NSL). Sign languages, like spoken languages, mark referential shift grammatically with a shift in deictic perspective. In addition, sign languages can mark the shift with a point or a movement of the body to a specified spatial location in the three-dimensional space in front of the signer, capitalizing on the spatial affordances of the manual modality. We asked whether the use of space to mark referential shift emerges early in a new sign language by comparing the first two age cohorts of deaf signers of NSL. Eight first-cohort signers and 10 second-cohort signers watched video vignettes and described them in NSL. Narratives were coded for lexical (use of words) and spatial (use of signing space) devices. Although the cohorts did not differ significantly in the number of perspectives represented, second-cohort signers used referential shift devices to explicitly mark a shift in perspective in more of their narratives. Furthermore, while there was no significant difference between cohorts in the use of non-spatial, lexical devices, there was a difference in spatial devices, with second-cohort signers using them in significantly more of their narratives. This suggests that spatial devices have only recently increased as systematic markers of referential shift. Spatial referential shift devices may have emerged more slowly because they depend on the establishment of fundamental spatial conventions in the language. While the modality of sign languages can ultimately engender the syntactic use of three-dimensional space, we propose that a language must first develop systematic spatial distinctions before harnessing space for grammatical functions.

## Introduction

Sign languages often exhibit a high degree of iconicity, compared to spoken languages, as signs and their referents exist in the same physical space (Taub, 2001). One arguably iconic component of sign languages is the use of distinct locations in signing space for grammatical purposes, such as locative marking and verb agreement (Klima and Bellugi, 1979; Supalla, 1982; Padden, 1983; Meier, 1987, 1990; Emmorey, 1996; Lillo-Martin and Meier, 2011). The present study explores the emergence of one class of grammatical devices, *referential shift* devices, that has been documented to include both lexical and spatial means to mark perspective changes (see Emmorey, 2002 for a review). We ask whether the earliest devices that emerge in Nicaraguan Sign Language (NSL) readily co-opted the iconic nature of space in the manual modality to mark shifts in reference.

When telling a story with multiple characters, a narrator must weave together a tapestry of information, integrating the perspective of the narrator with the perspectives of different characters to create a cohesive narrative. Signed and spoken languages alike employ a variety of referential shift devices to indicate multiple perspectives, and to mark when changes in perspective occur. To follow a narrative as it unfolds and to construct a mental representation of the event described, listeners rely on the narrator to provide information about the referents, their locations, their speech, and their actions. In spoken languages, narrators often use quoted speech to express different characters' points of view (Labov, 1972; Ochs, 1979; Schiffrin, 1981; Chafe, 1982; Tannen, 1982). English marks quoted speech with shifts in pronoun and tense, indicating a switched reference point for deixis. For example, in the sentence, “She said, ‘I need more paint,” the switch from the perspective of the narrator to the perspective of the character is marked syntactically by a shift from the third-person pronoun (*she*) in the matrix clause to the first-person pronoun (*I*) in the reported clause, and a shift from past (*said*) to present tense (*need*). Speakers can also rely on a shift in prosody to indicate quoted speech, changing intonation and voice quality to indicate something spoken by someone else (Clark and Gerrig, 1990). Because quoted speech is often not a faithful replication of exactly what was uttered at the moment of the speech act, but rather a reconstruction of what was said, this part of a narrative is sometimes called *constructed dialogue* (Tannen, 1986). Constructed dialogue can express not only a character's speech, but also his or her thoughts and feelings. If we change the matrix verb *say* in the above example to either *be all* or *be like*, (“She was like, ‘I need more paint”’) the quoted clause now indicates the character's internal thoughts (Blyth et al., 1990).

Like spoken languages, sign languages employ constructed dialogue, with a shift in deictic perspective, to express a character's speech and thoughts. Additionally, the manual modality allows signers to express a character's actions using a device known as *constructed action* (Liddell and Metzger, 1998). When representing actions, signers use their own bodies (*embodiment*) including the face, torso, and arms, to convey information about different characters and their actions. For instance, consider a story about a woman who enters a room and accidently lets the door close in the face of someone trying to enter behind her. A signer narrating this story could embody the characters' actions and reactions, first enacting closing a door with an expression of naïve ignorance, and then enacting bumping into a door and adopting an expression of surprise.

Importantly, the effective representation of multiple perspectives, through both constructed dialogue and constructed action, depends on clear and unambiguous marking of when there is a shift in reference. The narrator must clearly introduce the different characters, and when describing their speech, thoughts, and actions, unambiguously indicate which character's speech, thoughts, and actions are being expressed. If a narrator does not mark perspective shifts clearly, the listener may mistakenly attribute all of the speech, thoughts, and actions to a single character. As such, coherence and clarity in a narrative rely on the narrator's systematic use of lexical and grammatical cues to indicate who the referents are and when a shift in perspective occurs.

Referential shift devices similar to those found in spoken languages, such as pronominal shifts and pauses, have been identified in several sign languages, including American Sign Language (ASL), British Sign Language, Danish Sign Language, Swedish Sign Language, and South African Sign Language (Loew, 1984; Shepard-Kegl, 1985; Padden, 1986, 1990; Liddell, 1990; Lillo-Martin and Klima, 1990; Meier, 1990; Engberg-Pedersen, 1993; Poulin and Miller, 1995; Aarons and Morgan, 2003; Janzen, 2004; Cormier et al., 2013). Sign languages also leverage non-manual elements, signaling referential shifts through breaks in eye-gaze, head tilts, facial expression, and a *body shift* (Padden, 1986)^1^ from a neutral position to a specified spatial location associated with the referent (Engberg-Pedersen, 1993; Cormier et al., 2013). These devices differ from embodiment, where signers use the whole body or a part of the body to represent a particular character's body or its parts, in that these non-manual movements of the body signal a perspective change rather than convey information about a character's actions.

**Figure 1:**
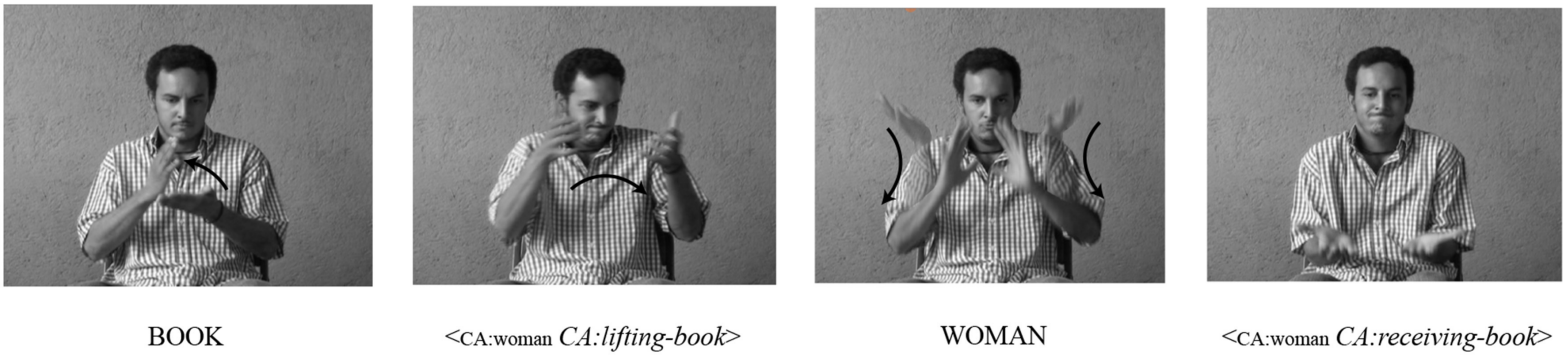
**An example of a spatially neutral *lexical label* as a referential shift device to mark perspective change in NSL**. The sign WOMAN in the third panel, produced in neutral space, is a lexical label marking a shift to the perspective of a new character^3^.

Referential shift devices in sign languages can be broadly categorized into two types of devices: *lexical* and *spatial*. The first category employs spatially neutral lexical devices to indicate a shift in referent. For instance, signers can assign a referent a *lexical label*, such as WOMAN^2^. Using that label, narrators can then introduce and re-introduce the character associated with the label, allowing the listener to understand when a shift to that character's perspective has occurred or is about to occur (for a review, see Cormier et al., 2013). An example of the use of the lexical label in NSL to indicate a shift in referent can be seen in Figure 1, where the signer represents the constructed actions of two different characters, one who lifts books down to the other, who receives them. Immediately before the second instance of constructed action, the signer produces the sign woman to indicate a shift in reference to the new character who receives the books. This referential shift device indicates that the agent of the receiving action is a different character from the agent of the previous lifting action.

**Figure 2:**
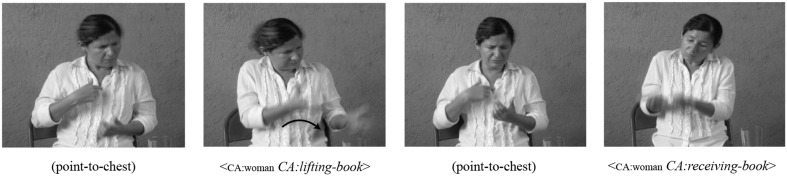
**An example of the *point-to-chest* to mark perspective change in NSL**. In the first panel, the point-to-chest, produced with the torso and shoulders in neutral position, indicates a shift to the perspective of the first character. In the third panel, a second point-to-chest marks a shift to the second character.

Prior work on referential shift in NSL has noted a second lexical device that has not yet been documented in other sign languages: a point to the chest (Pyers and Senghas, 2007). Using this device, signers point to themselves, explicitly indicating that they are about to take on the role of a character. This *point-to-chest* is sometimes, but not always, followed by a lexical label or a description of the character whose perspective the signer is about to adopt (e.g., the person with the books). The point-to-chest used in NSL in Figure 2 is distinct from the first-person pronoun used with referential shift in ASL in that the point-to-chest is produced before the shifted construction, signaling that the signer is about to change to the perspective of a particular character and construct the actions of that character, with a neutral positioning of the torso and shoulders, while the first-person pronoun in ASL is produced after a referential shift has been established, indicating self-reference by a character.

**Figure 3:**
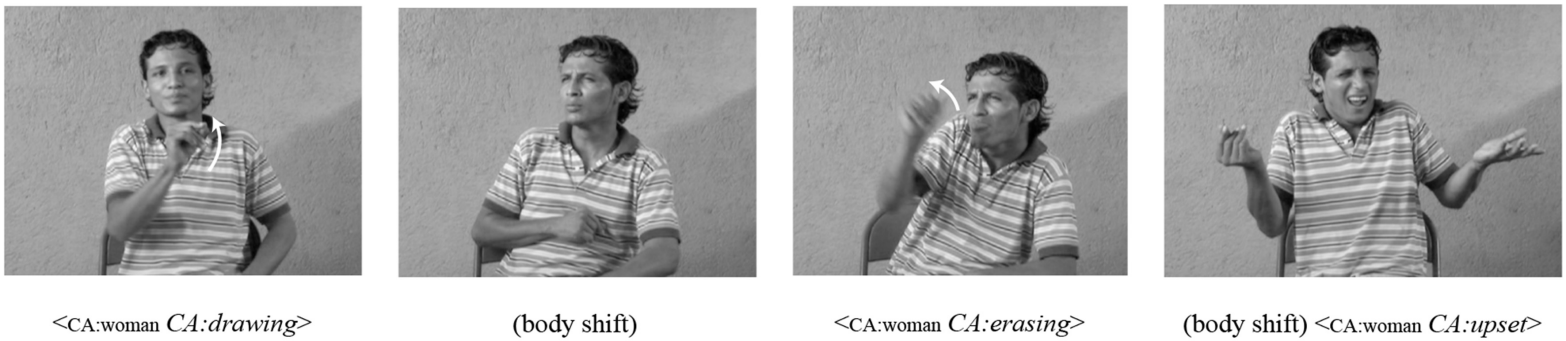
**An example of *body shift* as a referential shift device to mark perspective change in NSL**. The movements of the torso to the signer's left in the second panel, and to his right in the fourth panel, indicate referential shifts from one character to another.

**Figure 4:**
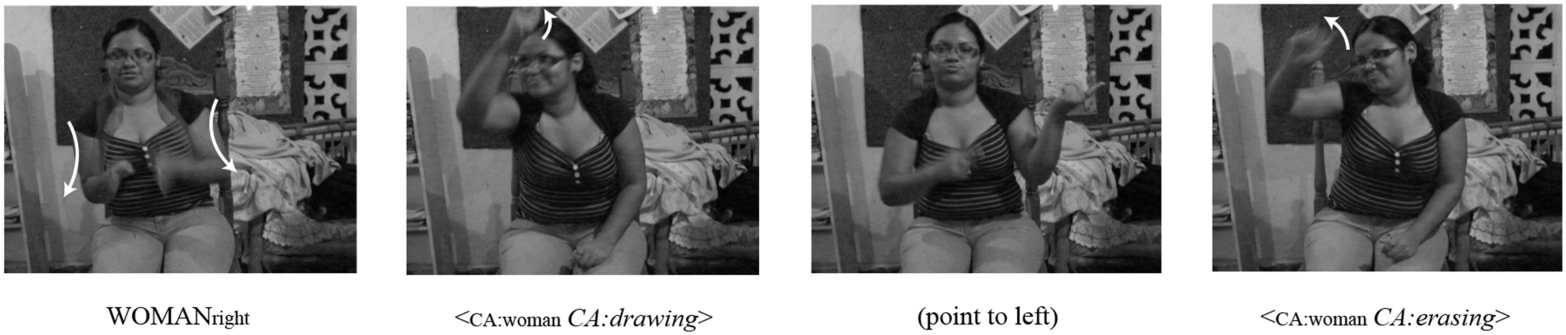
**An example of an *indexical point-to-space* as a referential shift device to mark perspective change in NSL**. The signer points to her left in the third panel to indicate a referential shift from one character to another.

**Figure 5:**
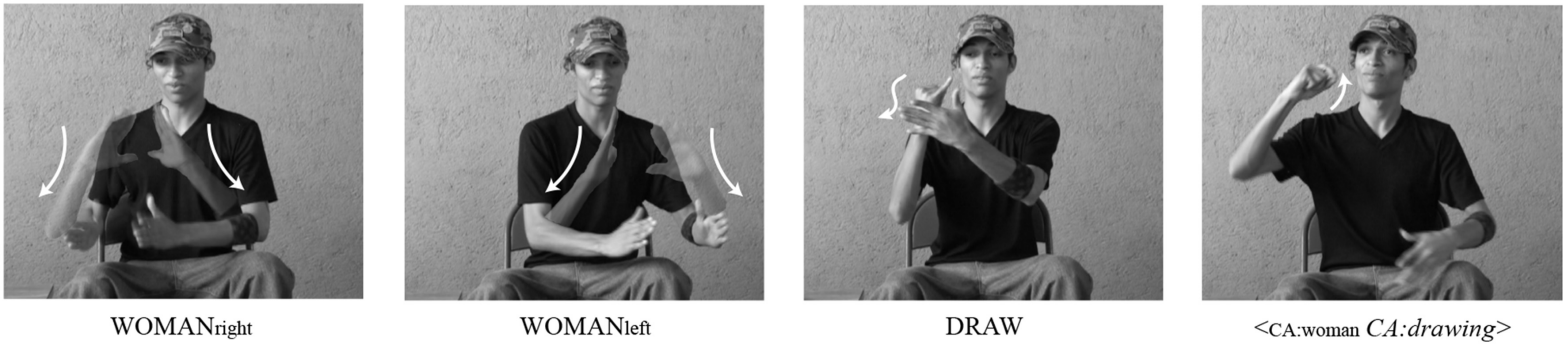
**An example of *spatially modulated lexical labels* in NSL**. In the first panel, the signer produces the sign WOMAN to his right, thereby associating the first woman with that location. In the second panel, he produces the sign WOMAN to his left, associating the second woman with that second location.

In contrast to lexical devices, *spatial devices* capitalize on the visual-spatial nature of sign languages and the ability of the signer to associate referents with locations in the three-dimensional signing space in front of the signer. For example, in many mature sign languages, specific locations in signing space are first associated with nominal signs. Once referents are associated with unique locations, the signer can anaphorically refer back to these locations, using direction of eye gaze, pointing, or a body or head shift. Examples of spatial devices in NSL are shown in Figures 3–**5**. In Figure 3, the signer uses a body shift along with his constructed action sequences to indicate the switch in perspective between two characters, one who draws on a whiteboard and another who then erases it.

Another spatial device to mark changes in perspective is an *indexical point to space* (Figure 4). Here the signer locates referents in different locations in the signing space, then points to one of these locations before engaging in constructed action in order to indicate a change in reference to the referent associated with that location in space.

A third spatial device in NSL is the *spatially modulated lexical label* (Figure 5). Here a lexical sign is produced in a specific location in the signing space, rather than in the neutral area in front of the signer's body. This device, like other spatial devices, can be used at first mention of a referent to establish a character in a narrative, or in later mentions in a narrative to shift reference to that character.

The use of space to indicate the locations of and relations among referents derives from the iconic relationship between the spatial representations in signing space and the true locations of the referents in the world (Emmorey and Reilly, 1995; Engberg-Pedersen, 1995; Taub, 2001). This iconic use of space is prevalent across mature sign languages, and has also been observed in gestural communication systems, called *homesigns*, that are developed by deaf children with their hearing family members when sign language is not available (e.g., Goldin-Meadow and Mylander, 1990; Engberg-Pedersen, 1993, 1995; Emmorey and Reilly, 1995; Coppola, 2002; Morgan et al., 2008). Bosworth and Emmorey (2010) suggest that the prevalence of iconicity may stem from the gestural origins of sign languages, perhaps due to the functional pressure for clarity and ease of communication. As such, during the emergence of a new sign language, when gestures and homesigns are reorganized into a structured language, one might expect to see creators of a new sign language readily avail themselves of the iconic nature of space to structure narratives.

**Figure 6:**
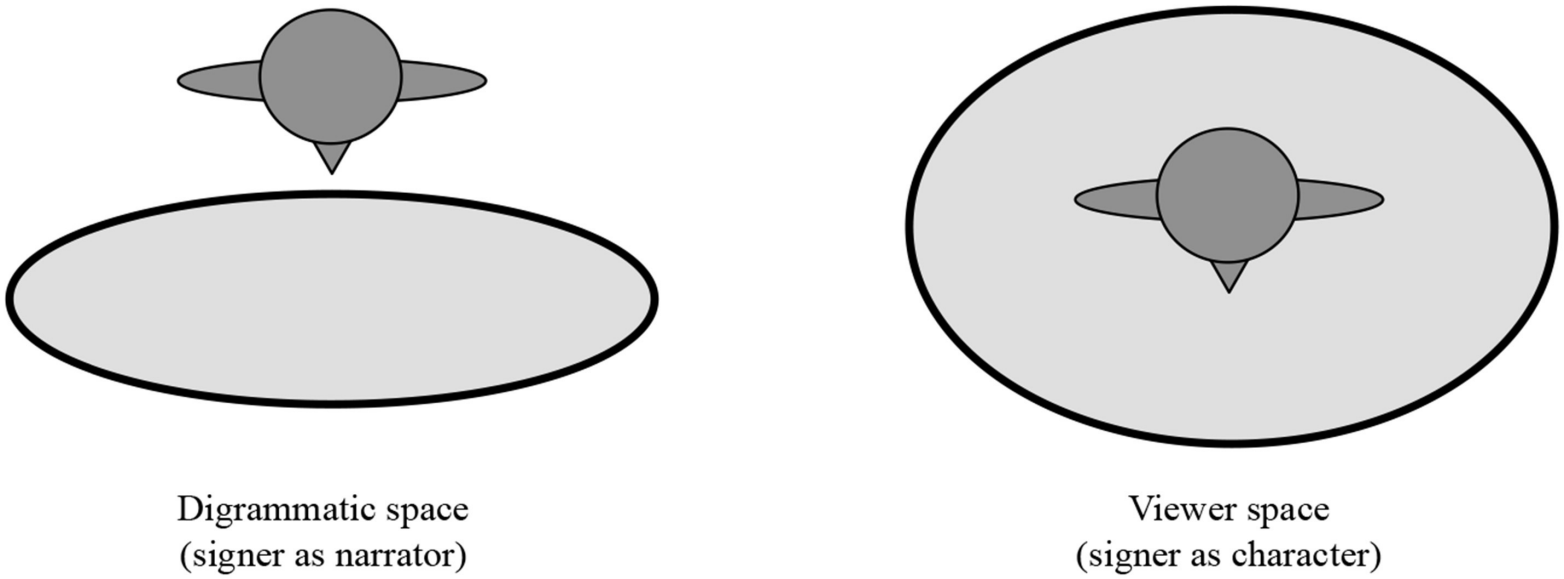
**Schematics of *diagrammatic space* and *viewer space* formats**. The dark gray figures represent the signer viewed from above, and the light gray oval represents the area of the signing space in which spatial signs are produced.

Because the signer's body can represent multiple characters and their different perspectives, as well as the signer's own perspective as the narrator, signers of mature sign languages generate different types of formats in their iconic representations of real-world spatial relations; these format types differ in perspective. One spatial format, *diagrammatic* space, situates the signer outside of the event, describing it from an observer's point of view (as in Figure 5). A second spatial format, *viewer* space, locates the signer within the event itself, describing it from an experiencer's point of view (as in Figure 3), and expressing any spatial relations relative to the character's first-person perspective (Emmorey and Falgier, 1999) (see Figure 6)^4^. Using these spatial representations, signers convey information about objects and characters, their locations, and spatial relations.

**Figure 7:**
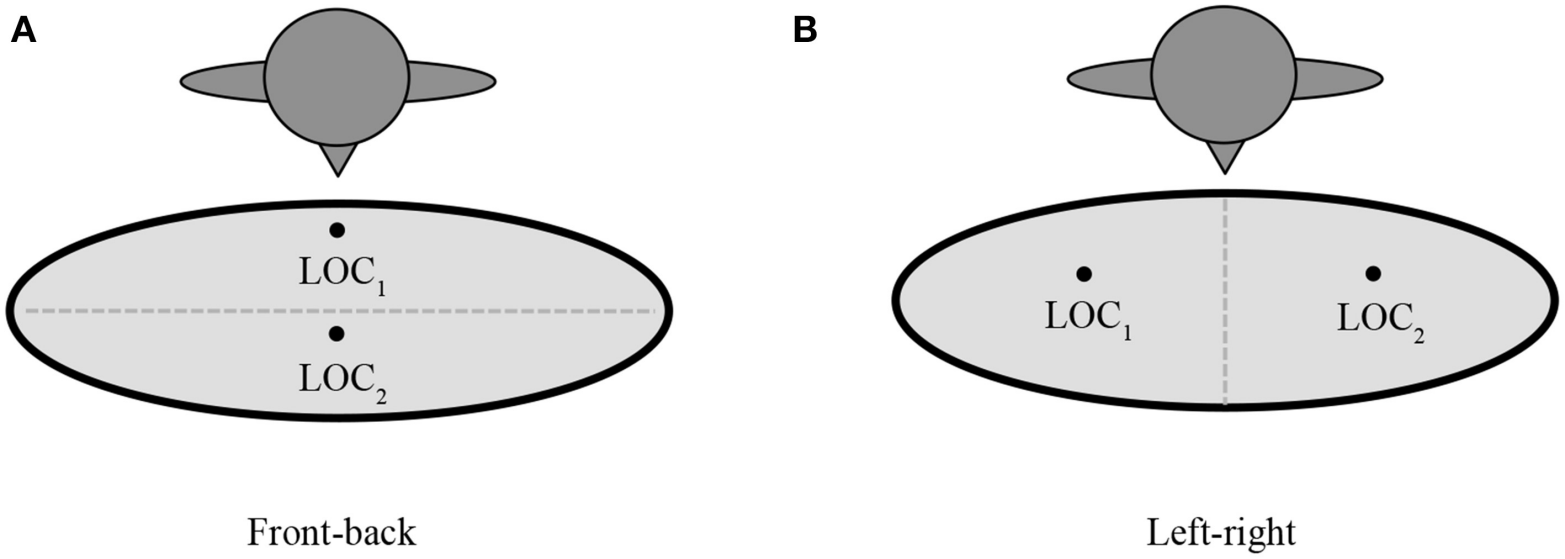
**Schematics of (A) front-back and (B) left-right spatial layouts within a diagrammatic space format**.

**Figure 8:**
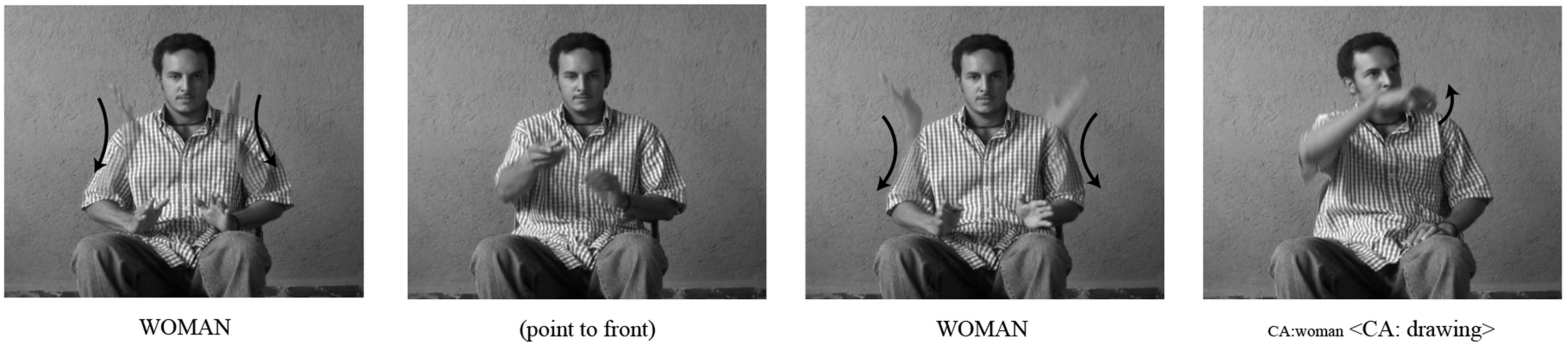
**An example of a front-back spatial layout within diagrammatic space in NSL**. The signer's point to a spatial location in front of the signer in the second panel associates that spatial location with the character of the second woman.

**Figure 9:**
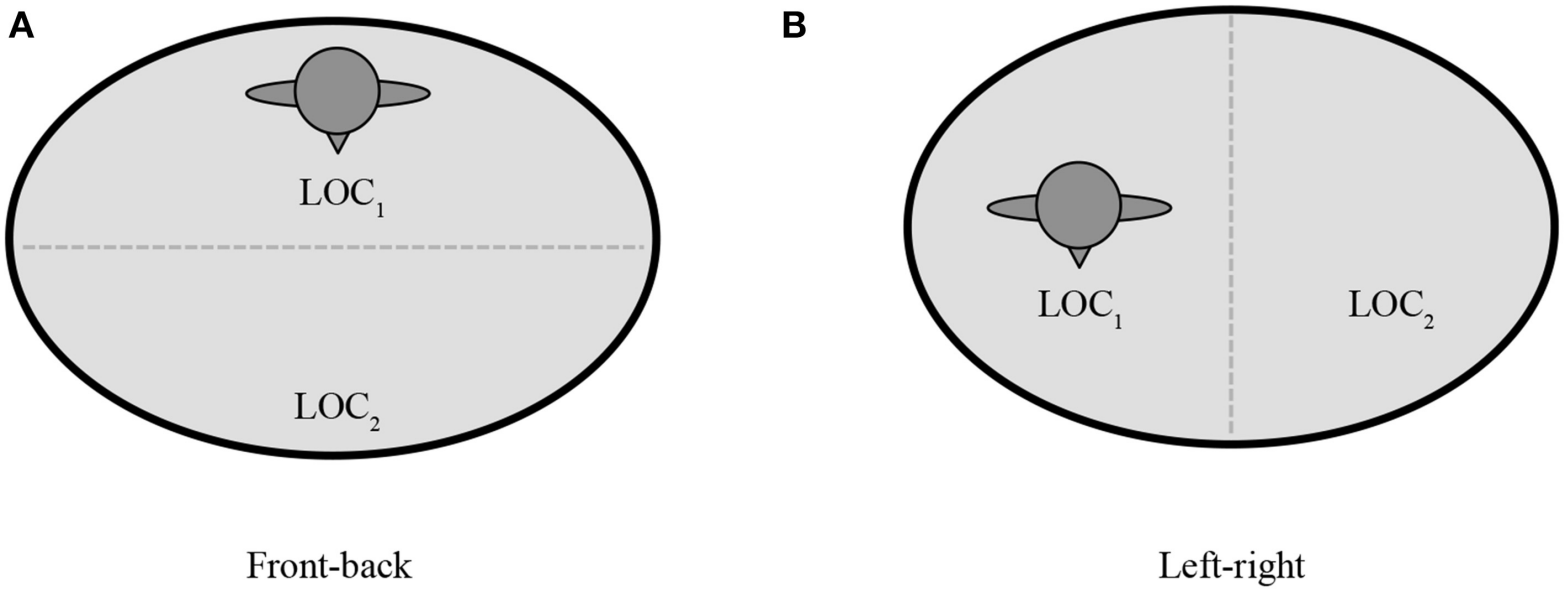
**Schematics of (A) front-back and (B) left-right spatial layouts within a viewer space format**.

Further, the signer has two options for representing the signing space (see Figures 7, 9): the signer can use the front-back axis or the left-right axis (Padden, 1986). When using left-right spatial contrasts, signers set up a referent on one side of the signing space (either left or right) and contrast it with a second referent set up on the opposite side of the signing space (Emmorey, 2002), as in Figure 5. With front-back spatial relations, signers tend to embody an animate character, and use the body as a locus with respect to which other characters or objects are assigned locations, in front of or behind the signer, as in Figure 8 (Emmorey, 2002; Perniss, 2007).

In mature sign languages, the preferred layout can depend on format. In ASL, diagrammatic space is typically used with left-right spatial contrasts, where the signer is narrating from the perspective of an observer, and contrasting referents are set up in front of the signer in the signing space. When using a viewer space format, where the signer is narrating from the perspective of a character, both left-right and front-back spatial contrasts can be used (Emmorey, 2002). These patterns have been found in ASL, a well-documented and mature sign language; we might expect to see different patterns of spatial layouts and formats cross-linguistically, and in a younger, emerging sign language.

Though the placement of referents in signing space is often iconic and derives its structure from the relative locations of objects in the world, spatial signing is not necessarily transparent (Emmorey and Reilly, 1995). As a signer narrates a story, the listener must construct a mental representation of the event, relying on the spatial information presented by the signer. As the narrative unfolds, the listener must continually map new information onto the developing mental image (Givón, 1995; Gernsbacher, 1997). Thus, narrative comprehension in sign languages is partly dependent on the signer's establishment and maintenance of the distinct spatial relationships among the referents throughout the narrative, particularly for short narratives of a specific event or situation^5^, such that the listener knows which referent's speech and actions are being represented (Winston, 1991). With such spatial consistency a subtle shift of the body or head relative to a spatial location associated with a referent can be sufficient to communicate a change in perspective (Emmorey, 2002).

To appreciate why setting up referents in spatial locations is crucial for narrative coherence, consider a signer who uses the body to represent multiple perspectives, via constructed action, but does not overtly mark when perspective changes occur. In such a situation, the listener may correctly understand that the narrator is telling a story about, say, one man who was walking and another man who was eating. Alternatively, the listener could easily misinterpret the account to be that a single man was walking and eating. Lexical devices could disambiguate which characters performed specific actions, without providing spatial information that reveals how the characters are located relative to one another. However, for spatial devices to be understood correctly, the signer must consistently map the different referents to contrastive locations in the signing space, across multiple signs, including constructed action sequences. As such, signers sometimes use non-spatial explicit referential shift devices such as lexical labels alongside spatial devices such as a body shift to make the referent more salient than it would be with the spatial device alone (Cormier et al., 2013).

The present study examined the origins of these complex grammatical systems. We follow the development of spatial and lexical grammatical devices for marking referential shift in an emerging sign language. By examining the early stages of a young sign language, we asked whether the richness of the spatial iconicity prevalent in the manual modality motivates early emergence of spatial devices in a new sign language.

The language under consideration emerged over the past four decades in Managua, the urban capital of Nicaragua. Prior to the 1970s, there was no established sign language in use in Nicaragua, but special education reforms in the late 1970s and early 1980s brought about drastic changes. With the opening of a primary school for special education, followed by a vocational center, deaf children and adolescents were able to socialize in greater numbers than ever before, giving rise to the birth of a new language (Kegl and Iwata, 1989; Polich, 2005). An initial group of fifty signers passed on their developing language to waves of new children entering the community each year, who, in turn, continued to add to the language's complexity and development (Senghas, 1995; Senghas and Coppola, 2001). By comparing the language of that initial *first cohort* of signers to that of those who entered in the language's second decade, the *second cohort*, we can see how the language has changed and grown.

The recent emergence of NSL offers the opportunity to examine when referential shift devices emerge in a language, which devices emerge first, and whether early-emerging devices differ from later-emerging devices in their use of space. We investigated the distribution and frequency of use of specific referential shift devices in NSL, and the pattern of device use between cohorts. If the creators of a new sign language can immediately harness the iconic nature of three-dimensional space, one might expect to see early emergence of spatial devices to consistently mark referential shift in the first cohort of Nicaraguan signers.

## Material and methods

### Participants

Eighteen deaf Nicaraguan signers participated in the study, ranging in age from 21.4 to 40.0 years. We grouped participants into two cohorts according to the year they were first exposed to NSL when they entered the primary school for special education (Senghas, 1995): 8 first-cohort signers (5 M, 3 F, *M_age_* = 33.1 years) were exposed to NSL before 1986, and 10 second cohort signers (6 M, 4 F, *M_age_* = 24.0 years) were exposed to NSL between 1986 and 1990. All participants were exposed to NSL by the age of 6 (Cohort 1 mean age of exposure: 4.6 years; Cohort 2 mean age of exposure: 4.0 years). All participants gave consent to participate and be videotaped as part of this study, and all were paid for their participation. The research protocol was approved by the Barnard College Institutional Review Board for the Protection of Human Subjects in Research and by the Wellesley College Psychology Department Research Ethics Committee.

### Materials

Participants were shown six video vignettes, presented as QuickTime movies on a laptop computer. The vignettes were 10–30 s in duration, depicting simple events that included two or three characters performing straightforward actions with no dialogue (Table 1). The vignettes were designed to eliminate the need for participants to make inferences about internal states to understand the actions depicted. Accordingly, the actions performed by the characters did not imply any hidden beliefs or intentions. For example, signers could (and often did) produce descriptions of Video #3 like, “The woman on the left was drawing on a whiteboard” (see Table 1). Of course, participants' responses could include descriptions of the characters' emotions or mental states; narrative information that goes beyond what is said is often a part of constructed dialogue.

**Table 1:** **Stimulus characteristics**.

**Video #**	**Description**	**Number of perspectives, including the narrator's**
1	Two women; one seated on a stool; the other approaches her and dumps a wastebasket full of paper over her head	3
2	Two women; one gives the other three stacks of books to hold	3
3	Two women; one draws on a whiteboard while the other erases her drawing	3
4	Two women; one throws a ball to the other, who holds a stack of papers, and drops the stack to catch the ball	3
5	Two women; both race to sit on a stool; one succeeds and knocks the other to the floor	3
6	Three women; one is standing inside a room, a second knocks on the door and the first lets her in; a third woman attempts to enter the room as the door is closing, but the door closes on her face, seen through a glass window in the door	4

### Procedure and coding

Participants watched each vignette and were instructed in NSL to “describe [to another signer from their cohort] what you saw.” Participants were permitted to watch the movies as many times as they liked. Narratives were videotaped (30 fps) for coding offline. Elicited narratives were coded by the first author, who is a fluent signer of ASL with 6 years of research experience with NSL, for (1) average length of the narrative, (2) proportion of perspectives represented, (3) use of space to assign spatial locations to referents, and the spatial format and spatial layouts used, (4) overt marking of referential shifts, and (5) types of referential shift devices used.

The average length of each narrative was computed in seconds. The counter started from the moment the signers lifted their hands until they dropped their hands, signaling the end of the narrative. The length of the narrative was calculated to check whether a greater number of perspectives represented might be a simple consequence of longer narratives.

Because the number of characters in the vignettes varied (see Table 1), we calculated the proportion, rather than the sum, of perspectives represented. This proportion was defined as the sum of perspectives represented by the signer divided by the total number of possible perspectives included in the narrative (the number of characters in the narrative plus the signer's perspective as the narrator; Table 1). Signers were coded as having represented the narrator's perspective if the signer included commentary or descriptive information from the signer's own perspective. Signers were coded as having represented a given character's perspective if the signer engaged in constructed action representing that character's perspective, such as imitating the facial expression, body posture/orientation or actions of the character, with a maximum score of 1 for each character, regardless of how many times that character's perspective was represented. For instance, if the signer was describing Video #3 and imitated the action of erasing a whiteboard, that sequence was coded as constructed action, representing the perspective of the character who did the erasing. Crucially, credit was given for representing a character's perspective even if the signer did not grammatically mark the perspective shift. Indeed, to someone naive to the video stimuli, many of the representations of the actions of multiple characters were produced without such marking, and could consequently be misinterpreted as multiple actions by a single character. For example, a listener might interpret a signed narrative with instances of constructed action of drawing and erasing either as a single character who is drawing and then erasing her own picture, or as two different characters, one who draws a picture and one who erases it. We included this measure of the number of perspectives represented to capture only whether participants explicitly encoded and expressed the actions of the different characters.

In addition, we coded whether signers assigned spatial locations to the referents at the beginning of each narrative, and whether they implemented a diagrammatic or viewer spatial format. We further looked at whether signers used front-back or left-right spatial layouts when assigning spatial locations to referents.

Signed narratives were also coded for the use of five types of referential shift devices that have been previously observed in NSL (Pyers and Senghas, 2007): *lexical label*, *point-to-chest*, *body shift*, *indexical point-to-space*, and *spatially modulated lexical label* (see Table 2). *Lexical label* and *point-to-chest* are lexical, non-spatial devices that indicate shifting to a particular character's perspective (see Figures 1, 2). *Body shift*, *indexical point-to-space*, and *spatially modulated lexical label* are spatial devices that associate physical locations in the signing space with particular referents (see Figures 3–5). The use of a referential shift device was coded as positive whenever the signer employed the device before introducing or re-introducing a character's or the narrator's perspective, and when shifting between perspectives. We coded the use of these devices only if they were used to indicate a shift in the referent before the signer engaged in constructed action, using his or her body to represent the perspective of the character, expressing the character's actions or feelings.

**Table 2:** **Referential shift devices**.

**Type of device**	**Referential shift device**	**Description**
Lexical	Lexical label	The signer's use of a spatially neutral lexical label, such as a noun, to indicate a change in reference. The most common lexical label used was woman, as women were featured in all of the character roles.
	Point-to-chest	The signer's use of a point to the chest, with the torso and shoulders in a neutral position, to indicate a change in reference.
Spatial	Body shift	The signer's use of a shoulder/body shift in physical space to the right or left from a central axis to indicate a change in reference.
	Indexical point-to-space	The signer's use of a point to a location in space to indicate a change in reference to the referent associated with that location.
	Spatially modulated lexical label	The signer's use of a lexical label produced in a particular location in space to indicate a change in reference to the referent associated with that location.

We analyzed only whether a particular device was present in each narrative, not the frequency with which that particular device appeared in the narrative. For instance, if a signer used all five referential shift devices while describing one vignette, that narrative would receive a 1 for each type of referential shift device. In cases where the signer used multiple devices to mark a single referential shift, each type of device used was coded as present.

## Results

First, we considered the average length of signers' narratives in the two age cohorts to determine whether any difference in the number of perspectives represented might be a reflection of the amount of time spent describing the events. The average signing time did not differ significantly between cohorts [Cohort 2: 15.50 s, *SD* = 5.55; Cohort 1: 12.50 s, *SD* = 4.39, *t*_(16)_ = 1.29, *p* = 0.22, two-tailed]. Each vignette contained multiple characters, and we measured the proportion of characters whose perspectives were represented or mentioned by signers in their narratives. Both cohorts expressed the majority of available perspectives in each narrative (see Table 3 for means and standard deviations). There was a marginally significant difference between the two cohorts in the number of perspectives represented [*t*_(7.30)_ = 2.33, *p* = 0.07, two-tailed, adjusted for unequal variances], although a Levene's test showed that the first cohort signers were significantly more variable in their performance (*F* = 13.75, *p* < 0.01). This difference between cohorts in the number of perspectives represented was driven primarily by the inclusion of explicit marking of and shifts between the perspective of the narrator and of a character. Since the narratives were not based on first-hand accounts, and were always told from the perspective of an outside observer (that is, the signer as the narrator), we conducted an additional analysis comparing the proportion of perspectives represented aside from that of the narrator, and found no significant difference between cohorts [Table 3, *t*_(8.96)_ = 1.52, *p* = 0.20, two-tailed, adjusted for unequal variances, *F* = 9.71, *p* < 0.01]. According to this analysis, signers from both cohorts were similarly able to represent the perspectives of the characters in their narratives.

**Table 3:** **Proportion of perspectives represented, and proportion of narratives in which signers set up referents in space, and used front-back and left-right spatial layouts, by each cohort**.

	**Cohort 2 (*N*** = 10)	**Cohort 1 (*N*** = 8)
	**Proportions (*SD*)**	**Proportions (*SD*)**
Perspectives (narrator's and characters')	0.98 (0.04)	0.82 (0.22)
Perspectives (characters' only)	0.99 (0.02)	0.96 (0.06)
Assigned spatial locations	0.98 (0.05)	0.67 (0.43)
Diagrammatic space	0.47 (0.22)	0.21 (0.19)
Viewer space	1.00 (0.00)	0.96 (0.12)
Front-back	0.50 (0.18)	0.63 (0.21)
Left-right	0.88 (0.14)	0.52 (0.26)

*Standard deviations given in parentheses*.

Next, we examined whether signers differed in their assignment of spatial locations to referents at the beginning of each narrative. Due to the categorical nature of the dependent variables, and because we were conducting between-subjects analyses, we used logistic mixed effects regression with item (video) and subject as random effects, where the use of space to assign spatial locations to referents and the absence of use of space were entered as 1 and 0, respectively. In the model, we looked at the effect of cohort on whether signers assigned spatial locations when describing the videos. The predictor variable, cohort, was coded such that the first cohort (signers who entered the community prior to 1986) represented the baseline (the intercept). Positive and negative coefficients are interpreted with respect to this intercept value, where a positive coefficient (β) represents an increase in the likelihood of the second cohort using the dependent variable of interest, and a negative coefficient represents a decrease. Accordingly, we report the coefficient representing the second cohort, indicating the difference from the first cohort, followed by Wald's z-score.

There was no difference between the two cohorts in how many narratives included the use of space for assignment of spatial locations to referents (β = 3.52, *Z* = 0.00, *p* = 1.00). We then considered whether the two cohorts differed in their use of diagrammatic and viewer space in their narratives. Second-cohort signers used diagrammatic space significantly more than first-cohort signers (β = 3.10, *Z* = 3.84, *p* < 0.001), but signers from the two cohorts did not differ in their use of viewer space (β = 3.41, *Z* = 0.00, *p* = 0.99). Note that signers can and did use both spatial formats within a single narrative. We then considered the frequency with which the two types of spatial layouts, front-back and left-right, were used by signers from each cohort in their narratives. The two cohorts did not differ in their use of front-back spatial relations overall (β = −0.47, *Z* = −0.67, *p* = 0.50), but did differ significantly in their use of left-right distinctions (β = 3.60, *Z* = 4.69, *p* < 0.001). In other words, signers from the two cohorts did not differ in how often they used space to assign locations to referents overall, nor did they differ in their use of front-back spatial relations when taking on the perspective of a character in the event (locating the other character in the space in front of them), but they did differ in their use of diagrammatic space (locating the narrator outside the event as an observer) and their use of left-right spatial relations for their referents.

Finally, we analyzed the frequency and type of referential shift devices used in the narratives. The data were submitted to a logistic mixed effects regression with item (video) and subject as random effects, and use of a specific referential shift device was assigned a 1 and the absence of that referential shift device was assigned a 0. Second-cohort signers used referential shift devices to explicitly mark a shift in perspective in significantly more narratives than did first-cohort signers (see Table 4 for means and standard deviations, β = 3.17, *Z* = 2.75, *p* < 0.01). Crucially, we observed a difference between the two cohorts in how consistent they were as a group in marking referential shift. Five first-cohort signers used referential shift devices in at least five of the six narratives, while the remaining three first-cohort signers used them in three or fewer narratives, that is, half the time or less. In contrast, nine of the 10 second cohort signers in our study used referential shift devices in at least five narratives, and the remaining second-cohort participant used them in four of the six narratives.

**Table 4:** **Proportion of narratives that included each referential shift device, by each cohort**.

**Referential shift device**	**Cohort 2 (*N*** =10)	**Cohort 1 (*N*** =8)
	**Proportions (*SD*)**	**Proportions (*SD*)**
Perspectives grammatically marked	0.95 (0.11)	0.67 (0.30)
Lexical Devices	0.58 (0.50)	0.50 (0.51)
Lexical label	0.52 (0.50)	0.38 (0.49)
Point-to-chest	0.20 (0.31)	0.19 (0.29)
Spatial Devices	0.87 (0.34)	0.27 (0.45)
Indexical point-to-space	0.38 (0.32)	0.06 (0.09)
Body shift	0.75 (0.29)	0.17 (0.24)
Spatially modulated lexical label	0.38 (0.49)	0.10 (0.31)

*Standard deviations given in parentheses*.

In investigating the types of devices used to mark referential shift, we found that second-cohort signers used significantly more spatial devices (β = 3.38, *Z* = 5.57, *p* < 0.001) but not more lexical devices (β = 0.36, *Z* = 0.76, *p* = 0.45) than first-cohort signers. There was no significant difference between cohorts in the use of neutral lexical labels as a device (β = 0.58, *Z* = 1.46, *p* = 0.14) nor point-to-chest (β = −0.75, *Z* = −0.36, *p* = 0.72). We next looked at whether there were cohort differences in the use of the different types of spatial devices. Compared to the first-cohort signers, second-cohort signers used significantly more spatially modulated lexical signs (β = 1.78, *Z* = 2.79, *p* < 0.01), indexical points-to-space (β = 2.71, *Z* = 2.77, *p* ≤ 0.01) and body shifts (β = 3.96, *Z* = 4.05, *p* < 0.001).

## Discussion

The recent emergence of a sign language in Nicaragua offers us the opportunity to capture the creation and development of new grammatical devices. We followed the emergence of referential shift devices over the first two sequential age cohorts of NSL, paying particular attention to the degree to which signers leveraged the iconic use of space for this function. There are reasons to expect referential shift to take advantage of spatial iconicity from the outset. Spatial devices for referential shift have been found in many mature sign languages, and may turn out to be a sign language universal. Furthermore, if a sign language already incorporates highly embodied, iconic representations within constructed action to depict the behaviors of characters in a narrative, it seems a natural first step to refer to the relative spatial locations of those characters to mark a shift in perspective from one character to another.

We observed that both the first and second cohorts of signers of NSL easily represented multiple characters' perspectives, readily switched back and forth among these perspectives, and did not differ in the number of character perspectives represented in their narratives. Previous work has documented delays in false-belief understanding in first-cohort signers (Pyers and Senghas, 2009), which had made us sensitive to the possibility that first-cohort signers might not effectively represent the different perspectives within a story. We found, however, that this was not the case; members of both cohorts, with equal frequency, encoded and represented the different characters' roles in their narratives.

Where the cohorts differed was in the use of devices to explicitly mark the shift from one perspective to another. This grammatical marking of referential shift was significantly greater in the second cohort. Both cohorts expressed perspective change using referential shift devices at least some of the time, suggesting that the seeds for linguistic marking of perspective emerged early in the language. However, the first cohort was both less frequent and more variable in their marking than the second. While three of the eight first-cohort signers marked referential shifts in half or fewer of their narratives, it was rare for a second-cohort signer to perform a shift without explicitly marking it. The consistency observed across the second-cohort participants suggests that over the late 1980s, while they were still young, NSL became increasingly stable in the marking of perspective changes in a narrative.

Where referential shift did appear in the signing of the first cohort, it was primarily as a non-spatial, lexical device; spatial marking, though present, was used far less. In contrast, second-cohort signers used spatial devices significantly more than the first-cohort signers did. This pattern of findings suggests that the use of space to mark referential shift was somewhat slow to emerge, relative to lexical devices.

The later emergence of spatial devices to grammatically mark referential shift does not appear to be due to the lack of a productive use of spatial layouts in general, throughout the language. Signers from both cohorts used the signing space in a concrete, iconic way to assign spatial locations to referents, along both the front-to-back and left-to-right axes, in about half of their narratives. This frequency suggests that both kinds of layouts, and the explicit use of the three-dimensional signing space, have been available since the earliest years of NSL. As we move from the first to the second cohort, the assignment of referents to locations to the left and right increased. Interestingly, we did not see a similar increase in the use of the front-to-back axis. Evidently, as the language matured, the balance between the two layouts changed, favoring differentiation along the left-to-right axis, at least for this function.

Along with the change in spatial layout, we observed changes in the nature of the spatial format applied in the narratives. Both cohorts readily described events using a character's perspective, even multiple characters' perspectives, in viewer space. That is, signers from both cohorts adopted the perspective of a character within their constructed action utterances. However, the use of diagrammatic space to frame the event from the perspective of an outsider, here the narrator, increased across cohorts, occurring in less than a quarter of the first cohort's and about half of the second cohort's narratives. We suggest that these changes—the increase in the use of differentiation along the left-to-right axis, and the increase in the use of diagrammatic space to structure the narrative—follow from other changes in the language, specifically, (1) the establishment of conventions for conveying left-right spatial contrasts (*to the left of*, *to the right of*) and (2) the development of more complex story structures, framed at the level of a third-person narrator.

### The emergence of left-right spatial contrasts

Despite its apparent iconicity, the use of space to the left and right of a signer to convey the concept of physical spatial contrasts, such as *left of* and *right of*, is not automatic or transparent, and was not available at the outset of NSL. Descriptions of left-right relations are grounded in real-world space, and the mapping from real-world-space to signing-space can be ambiguous. Left-right contrasts present a particular challenge, because they fall along an axis of symmetry—the left side of the body is symmetrical to the right side—and because perspective differs from one persons' viewpoint to another. This combination makes them more subject to ambiguity than up-down and front-back contrasts. The convention in ASL and many other mature sign languages is that left-right spatial contrasts are typically described from the viewpoint of the signer (Emmorey, 1996; Pyers et al., 2008). Previous work on NSL has shown that second-cohort signers introduced consistency in the use of spatial language, systematically marking left-right spatial relationships, and linguistically distinguishing among contrastive locations within the signing space. The older, first-cohort signers do use the signing space to describe spatial relationships, but do not do so systematically, resulting in ambiguous spatial descriptions (Pyers et al., 2010). Specifically, first-cohort signers might use the same spatial locations to describe objects to their left as for objects to their right, while second-cohort signers would use distinct locations to the left and right side of the signing space to convey the relative locations of objects to the left and right side in the real world. Evidently this spatial contrast took some time to develop in NSL, and did not conventionalize until the first cohort had already reached adolescence. Once the language had established conventions for left-right spatial contrasts in descriptions of physical space, signers readily applied this distinction in devices marking abstract reference. In that sense, the spatial referential shift devices developed quickly.

### The development of the narrator within the narrative

Conventions at the level of discourse structure similarly took some time to emerge, and did not necessarily arise automatically once more local devices for sentence structure were available. In related work on the emergence of NSL, the use of devices for building discourse cohesion increased as the language was passed from the first to the second cohort. These developments included an increase in the range and frequency of devices for explicitly marking grammatical subjects (Coppola et al., 2013) and the development of anaphoric uses of pointing (Coppola and Senghas, 2010). Consequently, signers could manage narratives more explicitly at a meta-level, introducing and referring back to characters unambiguously. The introduction and maintenance of characters within a story is often performed at the discourse level of the narrator, across sentences. Increasing grammatical specificity at the local level (e.g., distinguishing subjects and objects) likely enabled more complex narrative structure, which in turn created a context in which any referential devices needed to be applied consistently to effectively maintain reference in longer utterances, including across sentence boundaries. That is, the development of more complex narratives may have created pressure to express multiple distinct perspectives unambiguously, and to explicitly distinguish the narrator's perspective from the characters' perspectives in a story. Lexical and spatial devices being used to disambiguate reference might have then been taken up as grammatical markers of referential shift.

### Similarities to other emergent systems

Work on the development of verb agreement in Israeli Sign Language (ISL), a language around 75 years old, shows a similar pattern of a shift from use of front-back space to left-right space as the language develops (Meir, 2012). The earliest inflected forms of agreeing verbs in ISL are produced on the front-back axis, where the argument is associated with a spatial locus in front of the signer, and the directionality of the sign moves from the signer's body to the locus. In later forms, arguments are associated with spatial locations to the left or right of the signer, and inflected signs are produced along a left-right axis, originating from the signer's body or a spatial location off the body and ending at the locus associated with the object or recipient argument.

In contrast, Al-Sayyid Bedouin Sign Language (ABSL), an emergent village sign language that is approximately the same age as ISL, has not yet developed ways of using space in this grammatical way, and lacks a spatial verb agreement system (Meir et al., 2007). This later, or absent, grammatical use of the signing space may be indicative of differences between deaf community sign languages, like ISN and NSL, and village sign languages like ABSL (see Meir et al., 2010). Village sign languages typically have a smaller number of deaf members—in this case, ABSL has about a tenth the number of deaf members as NSL—with a correspondingly higher degree of shared information (Sandler et al., 2005). The resulting greater intimacy within the community may put less functional pressure on the language to make the kinds of grammatical distinctions we document in NSL. We would predict that any future development of a grammatical use of space in ABSL would include viewer-space perspective and front-back contrasts emerging first, and diagrammatic-space perspective and left-right contrasts appearing later.

Note that the changes we explore here do not represent a simple increase or decrease in spatial iconicity, but rather, a change in the nature of the spatial mappings that different grammatical devices exploit. We can identify two types of iconicity in use, which map on to different aspects of events. The first type, which includes enactment, depicts the actions of agents from the agent's own perspective. The second type, which includes diagrammatic-space depictions and spatial body shifts, depicts the actions and locations of agents from a narrator's perspective. These two types of iconicity use mutually incompatible mappings. For example, through enactment, a signer can faithfully replicate the behaviors of the referent, using the movement of the signer's body to represent movement of a referent's body. But when the signer moves the body to indicate a shift of reference, that body movement no longer maps to the movement of the referent's body. There is no corresponding movement, by any referent, that occurs in the actual event. The first type of iconicity, enactment, is highly effective in portraying the actions of a single character, but is limited in its capacity to depict other components of an event, such as other referents and their actions. Conversely, spatial iconicity captures the relationship among the components of the event. Once you have both types of iconic representations in use in a language, grammatical devices are necessary to effectively switch back and forth between the two.

### Parallels to acquisition

In some ways, the developments we have documented in the emergence of NSL parallel the acquisition of language by children. Previous research has found that a consistent and effective use of spatial contrast develops relatively late in native-signing children's acquisition of mature sign languages (Schick, 1990). Children acquiring ASL are able to express the perspective of another character starting from age three, but initially do so using direct quotation and constructed action within embodied representations, in which the signer's body represents the body of the referent character. At about 5 years of age, native signers are able to establish and use consistent spatial locations for co-reference and verb agreement (Loew, 1984; Lillo-Martin, 1991). It is not until 7–10 years of age that signers fully master anaphoric and other “long-distance” uses of space, applying spatial loci consistently across a set of utterances to produce cohesive narratives (van Hoek et al., 1987, 1989; Bellugi et al., 1990; Emmorey, 2002). Though the use of space is clearly a fundamental aspect of mature sign languages, utilizing signing space to encode and maintain reference throughout a narrative is a complex and late-developing skill.

The development of the narrative skill required to express a narrator's perspective and that of multiple characters is similarly gradual and relatively late. Switching among these perspectives requires both cognitive maturity and linguistic skill, and children acquiring spoken language typically master it only in the middle-school years (Berman and Slobin, 1994). This protracted development may inform why, along with their overall less frequent use of referential shift devices, first-cohort signers frequently produce narratives situated from the perspective of a character, using first-person embodiment devices, rather than structuring the story from a narrator's perspective, even though the narrator perspective most closely resembles their own.

As we consider these parallels between sign language acquisition and emergence, it appears that a primary, or more basic representation of perspective is generated within an embodied, viewer-space format, with spatial contrasts along the front-to-back axis. Yet most, if not all, mature sign languages actively use a diagrammatic format, and use contrasts along the left-to-right axis. These conventions clearly are taking hold in NSL; indeed, by the second cohort, the left-to-right axis has become the preferred one for spatial contrasts. Why might a language change in this way? If we may speculate, the left-to-right axis might offer advantages in perceptual salience that enable signers to better exploit the three-dimensional signing space. Signing space is used for a variety of grammatical functions, such as verb agreement, anaphora, and other types of co-indexation, which utilize non-manual as well as manual sign elements to identify particular locations near the signer's body. Signers can associate locations with particular referents, and then use pointing, eye gaze, and subtle movements of the head or torso relative to those locations to refer back to those referents (Thompson et al., 2006). Discriminating between less overt markers such as eye gaze and body movements may be easier with contrastive locations along the left-to-right axis than locations along the front-to-back axis. In other words, a glance to the left may be easier to discriminate from neutral eye-gaze than a glance forward. Moreover, the physical signing space is wider left-to-right than it is long front-to-back, allowing for a greater number of distinct locations. Since a signer cannot easily refer to locations behind the back, the use of the front-to-back axis realistically offers only one location in contrast to the signer's body. Thus, the left-to-right axis allows for more contrastive locations that are more easily distinguished.

Despite these advantages, there is a cost in adopting the left-to-right axis for spatial contrasts. Because movements to the left and right are symmetrical, they may be more difficult to encode or remember. Research in spatial cognition has found that people can differentiate and recall contrasts along asymmetrical axes, such as up vs. down and front vs. back, better than symmetrical ones like left vs. right (Franklin and Tversky, 1990; Bryant et al., 1992). Furthermore, the contrast between left and right depends on perspective. When talking about the location of non-jointly viewed locations in physical space, the signer's right can correspond to the listener's (or a character's) left. This ambiguity may explain why it takes time for a community to converge on conventions that use symmetrical relations for contrasts in reference.

Our examination of the emergence of referential shift devices in NSL has revealed that grammatical conventions for indicating shifts in perspective emerged over two sequential age cohorts of signers, who learned the language in its first two decades. The first cohort had a fair amount of variability in their production, but even so, their narratives already contained the seeds of lexical and spatial elements that would become more frequent, and possibly obligatory, in the language of the second cohort. Spatial devices appear to have emerged more slowly, but have recently become as prevalent as non-spatial, lexical devices. Previous work on NSL shows that second- but not first-cohort signers use consistent spatial language for other functions, and that the use of space to systematically assign semantic roles to the arguments of verbs emerged only with the second cohort. Spatial referential shift devices may have emerged later because they depend on the establishment of fundamental spatial conventions in the language. We conjecture that the systematic use of spatial devices in more local environments, such as within phrases and sentences, allowed them to be repurposed at the discourse level. Thus, while the modality of sign languages can ultimately engender the syntactic use of three-dimensional space, we propose that a language must first develop consistent and systematic local spatial contrasts before harnessing space for long-distance, abstract grammatical functions. The consistent use of spatial language and the grammatical use of space for shifting reference did not spring up unaided in first-cohort signers. Rather, the second cohort of signers, as children, built upon the achievements of the first. In this way, two sequential age cohorts of children transformed Nicaraguan signing from its gestural seeds to the full, complex language it is today.

### Conflict of interest statement

The authors declare that the research was conducted in the absence of any commercial or financial relationships that could be construed as a potential conflict of interest.

## Footnotes

^1^This type of role-shifting, where there is a change in body position to indicate a shift in perspective, has been termed *contrastive role-shifting*. Padden (1986) argues that in contrastive role-shifting, at most only two roles can be contrasted. In the case of narratives with more than two referents, the third referent is introduced in a different (subordinate) role-shifting structure. The subordinate structure (the third referent and one of the original two referents) is contrasted with the initial contrastive role-shift structure (first and second referent). Importantly, the contrastive role-shifting structures are still associated with separate spatial locations.

^2^English glosses for signs appear in SMALL CAPS.

^3^Following the conventions in sign linguistics literature (see Emmorey, 2002; Cormier et al., 2013), a token of constructed action is indicated with “CA:” followed by a description in lower-case letters. The concepts described with CA are italicized. When a referent is specified, CA:x is used, where x is the referent, with angled brackets placed at the beginning and end of the CA.

^4^Other terminology for these two spatial formats include observer perspective vs. character perspective (Perniss, 2007), depictive vs. surrogate space (Liddell, 2003), and narrator vs. protagonist perspective (Slobin et al., 2003).
